# Laryngo fiberscopy-guided suspension procedure for an ectopic lingual thyroid obstructing airway

**DOI:** 10.1186/s40792-018-0531-3

**Published:** 2018-09-19

**Authors:** Tsubasa Aiyoshi, Toshihiro Muraji, Isho Izumi, Miki Toma, Kazuto Suda, Toshihiro Yanai, Kouji Masumoto

**Affiliations:** 1 Department of Pediatric Surgery, Ibaraki Children’s Hospital, Mito, Ibaraki Japan; 2 Department of pediatrics, Ibaraki Children’s Hospital, Mito, Ibaraki Japan; 3 Department of Pediatric Surgery, Faculty of Medicine, University of Tsukuba, Tsukuba, Ibaraki Japan

**Keywords:** Ectopic thyroid, Lingual thyroid, Airway obstruction, Surgery, Transposition

## Abstract

**Background:**

Currently, there is no consensus regarding the optimal therapeutic strategy for the management of an ectopic lingual thyroid. A surgical approach is suggested when airway obstructive symptoms cannot be tolerated at all, or when bleeding or malignancy occurs. However, for patients in whom ectopic thyroid is the only functioning thyroid tissue, complete surgical excision needs to be followed by lifelong hormone replacement therapy. We report the case of an infant with ectopic lingual thyroid obstructing the airway that was treated using our novel surgical procedure.

**Case presentation:**

A 10-day-old male infant presented with symptoms of airway obstruction and subclinical hypothyroidism. Imaging tests revealed an ectopic lingual thyroid and the absence of a normal pretracheal thyroid gland. We administered oral levothyroxine to lower his thyroid stimulating hormone (TSH) level and reduce the volume of the lingual mass; however, his airway symptoms did not improve. Subsequently, we performed a surgical intervention when he was 2 months old. We split the hyoid bone, and then suspended the lingual thyroid by suturing it to the hyoid bone to elevate the epiglottis. We confirmed the degree of suspension using intraoperative laryngo fiberscopy. After the surgery, the symptoms of airway obstruction were resolved and the patient was clinically euthyroid on low-dose oral levothyroxine.

**Conclusions:**

Our laryngo fiberscopy-guided suspension procedure can be an effective surgical procedure for the treatment of ectopic thyroid. This relatively simple surgical procedure could completely preserve the patient’s thyroid tissue and resolve airway obstruction.

## Background

An ectopic thyroid gland is caused by failure of descent of the thyroid gland anlage early in the course of embryogenesis. The lingual region is the most common ectopic location of the thyroid gland, and this anomaly can cause airway obstruction by compressing the epiglottis. Surgical approaches, such as resection of the mass, are considered for patients with significant airway obstruction, or in those with bleeding or malignancy. Patients in whom the ectopic thyroid tissue is the only functioning thyroid tissue, careful preservation of the secretory function of the ectopic thyroid is required to avoid total loss of thyroid function, particularly in pediatric patients. We report a case of ectopic lingual thyroid in such a pediatric patient in whom a novel laryngo fiberscopy-guided suspension procedure was performed to preserve the ectopic thyroid gland.

## Case presentation

**Fig. 1:**
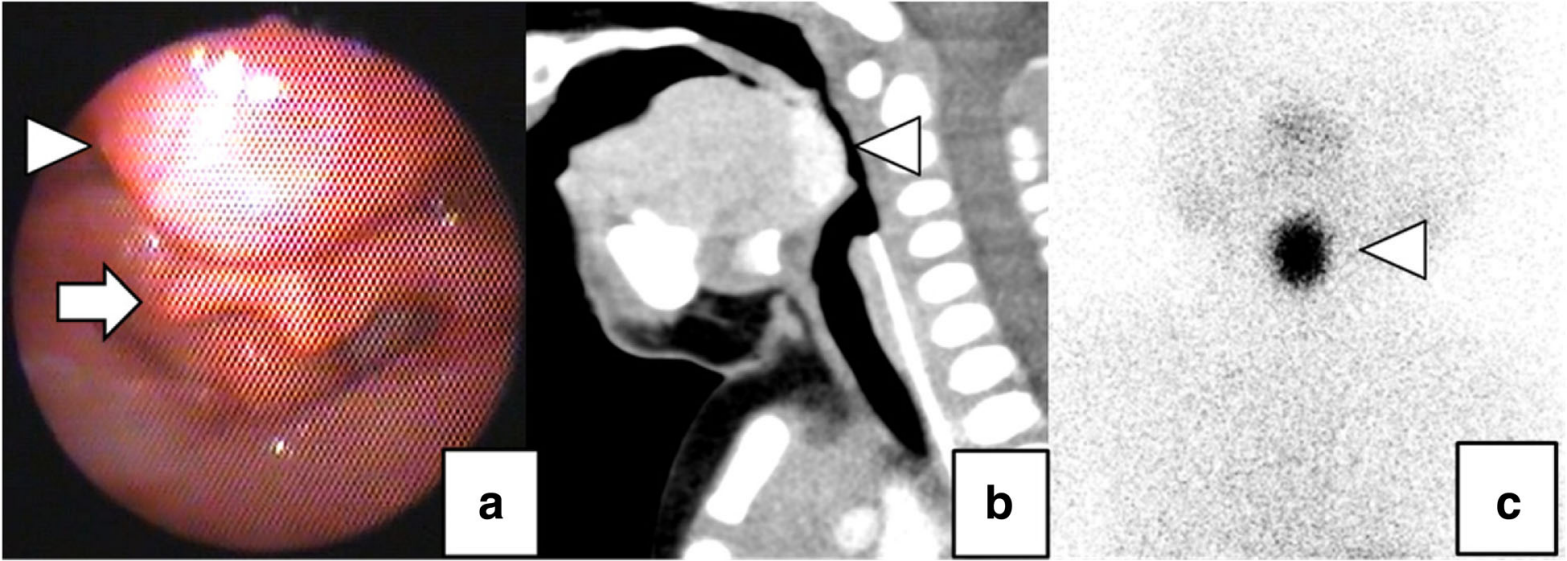
Preoperative imaging tests. Laryngeal endoscopy revealed a lingual mass (arrow head) compressing the epiglottis (arrow) (**a**). Enhanced CT (**b**) and thyroid scintigraphy using iodine-123 (**c**) revealed that the mass (arrow heads) was an ectopic thyroid and that there was no normal pretracheal thyroid gland

**Fig. 2:**
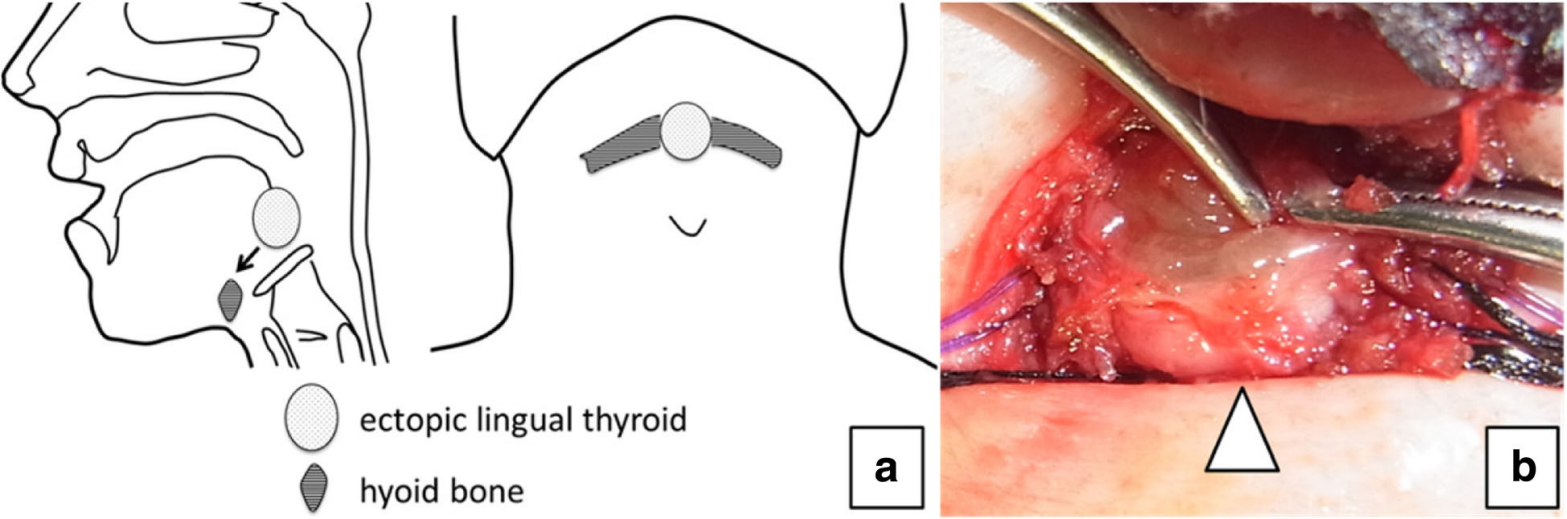
Mass suspension procedure for lingual ectopic thyroid. Schema of mass suspension procedure (**a**). We suspended the mass (arrow head) by suturing it to the hyoid bone (**b**)

**Fig. 3:**
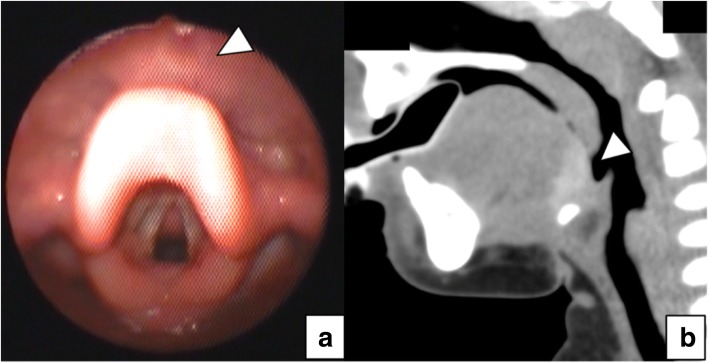
Postoperative imaging tests. Laryngeal endoscopy performed 6 months postoperatively (**a**) revealed the disappearance of airway compression by the lingual mass, and CT performed 8 months postoperatively (**b**) revealed relocation of the lingual thyroid gland towards the hyoid bone

A 10-day-old male infant was referred to our hospital because of suspected congenital hypothyroidism. The patient presented with symptoms of airway obstruction, such as an inspiratory stridor and retracted breathing. A hormonal test revealed subclinical hypothyroidism with a free thyroxine level (1.44 ng/dL) within the reference range, although the thyroid stimulating hormone (TSH) level (34.6 μIU/mL) was increased beyond the normal range. Laryngo fiberscopy revealed a lingual mass compressing the epiglottis (Fig. 1a). Enhanced computed tomography (CT) and thyroid scintigraphy revealed that the mass was an ectopic thyroid with the absence of a normal pretracheal thyroid gland (Figs 1b, c). The patient received oral levothyroxine at a dose of 12 μg/kg/day for 4 weeks to lower the TSH level and reduce the volume of the ectopic thyroid tissue. However, we observed no reduction in the volume of the thyroid tissue and a concomitant progression in his symptoms of airway obstruction. He underwent surgery to relieve the airway obstruction when he was 2 months old. Under general anesthesia, nasotracheal intubation was performed in a sniffing position, and a transverse skin incision measuring 2.5 cm was made at the level of the hyoid bone. We split the hyoid bone at the midline, dissected the base of the tongue towards the foramen cecum, detected the ectopic thyroid mass, and suspended the mass by suturing it to the hyoid bone (Fig. 2). We used 5–0 monofilament absorbable sutures and sutured between the lingual thyroid and the hyoid bone. The bite length of both the lingual thyroid and hyoid bone was about 3 mm. The points of suturing were to the lateral side of the lingual thyroid and to the front of it. The total number of suture threads was 3. The degree of suspension of the ectopic thyroid was guided by an intraoperative laryngo fiberscopy to confirm the complete elevation of the epiglottis. The patient was not extubated until postoperative day 4 and needed noninvasive positive pressure ventilation until postoperative day 22. Laryngo fiberscopy performed 6 months postoperatively revealed the complete disappearance of compression of the epiglottis by the lingual mass, and CT performed 8 months postoperatively also revealed the relocation of the lingual thyroid gland towards the hyoid bone (Fig. 3). When the patient was 2 years 6 months old, his height was 94.1 cm(1.4 SD), weight was 14.0 kg(1.0 SD), free T3 was 2.97 pg/mL, free T4 was 1.48 ng/dL, and TSH was 4.178 μIU/mL. He was taking daily levothyroxine 4.5 μg/kg/day and had been kept in the euthyroid state. Since we were able to preserve his thyroid gland (which is his only functioning thyroid tissue), the postoperative control of his thyroid hormone status was relatively easy. The surgery was complicated by the development of a salivary fistula that was spontaneously resolved 5 months postoperatively.

## Discussion

The thyroid gland attains its expected normal anatomical position in the pretracheal region by migrating caudally from the foramen cecum at the base of the tongue during the seventh week of fetal life. Ectopic thyroid tissue occurs because of incomplete migration. Clinically, the incidence of lingual thyroid is 1 in 100,000 cases [1]. This anomaly is 4 times more common in women than in men. Although it can occur at any age, it manifests more commonly during childhood, adolescence, and around the time of menopause. This could be attributed to the increased demand for thyroid hormones during these stages, which is met by increase in the level of circulating TSH through growth of ectopic thyroid tissue [2]. In approximately 90% of ectopic thyroid cases, the ectopic thyroid tissue is found at the base of the tongue as a lingual thyroid gland. In 75% of those presenting with a lingual thyroid, no other thyroid tissue is present [3].

Lingual thyroid is usually asymptomatic, unless accompanied by an increase in the size of the gland. Symptomatic patients present with dysphagia, dysphonia, a foreign body sensation in the throat, cough, pain, bleeding, and/or dyspnea [1]. Several previous studies have reported that approximately 33–62% of patients with ectopic thyroid develop hypothyroidism with increased TSH levels [2]. It has been reported that approximately 24% of children with primary nongoitrous hypothyroidism show an ectopic thyroid [4].

There is no consensus regarding the optimal therapeutic strategy for the management of such patients. Asymptomatic patients require a strict follow-up for the early detection of malignancy or the development of other complications [3]. For patients presenting with mild symptoms and a hypothyroid state, levothyroxine replacement therapy may be effective. Administration of a suppressive dose of thyroid hormones decreases TSH levels and can reduce the ectopic glandular volume and consequently reduce symptoms of compression [1, 5, 6]. However, there is no consensus about its dose or the duration. Radioactive iodine therapy has also been reported as a useful non-surgical treatment option for lingual thyroid. However, it should be avoided in children and young adults owing to potential harmful effects on the gonads and other organs [5].

Surgical intervention is warranted in patients with severe symptoms of airway obstruction, bleeding, and/or malignancy [3]. A total excision is the most common surgical procedure performed in such cases. Several surgical approaches have been described, such as transoral, transhyoid, suprahyoid, or lateral pharyngotomy [5]. Transoral CO2 laser excision and transoral radiofrequency ablation have been reported as mini-invasive surgical approaches [4, 7]. However, patients in whom the ectopic thyroid is the only functioning thyroid tissue, total surgical excision needs to be strictly followed by lifelong hormone replacement therapy. Regarding preservation of the patient’s own ectopic thyroid gland, several reports have been published on transposition or autotransplantation of the lingual thyroid instead of its total excision. In those cases, lingual thyroids were transposed or transplanted to the muscles of the neck, floor of the mouth, abdominal or pectoral regions, with or without a vascular pedicle flap. The procedures were performed via a transoral, transhyoidal, or lateral approach [8–13].

Surgical intervention necessitates careful and close attention to airway management because surgical access and excision are associated with a significant amount of edema formation. A preoperative tracheostomy should be considered in high-risk patients [4].

Our laryngo fiberscopy-guided suspension procedure is a novel surgical procedure for the management of a lingual thyroid. It involves suturing and fixation of the ectopic thyroid tissue to the hyoid bone. We adopted this procedure because total excision of the mass would not necessarily release the airway obstruction if laryngomalacia continues to cause the epiglottis to hang over the glottis. Its advantage is that it preserves the ectopic thyroid tissue, which therefore contributes to postoperative hormone replacement therapy and it can resolve an airway obstruction certainly, using intraoperative laryngo fiberscopy. Moreover, the surgical technique of our procedure is easier and simpler than that of other reported transposition or autotransplantation procedures. Pediatric surgeons are accustomed to performing the Sistrunk procedure for thyroglossal cysts via the transhyoidal approach. We developed the idea for present procedure from the Sistrunk procedure itself. Before the operation, we carefully considered the safety of the procedure, especially with respect to the management of the airway. Because we were attempting a minimally invasive surgery based on the Sistrunk procedure, we did not perform preoperative tracheostomy. Vocal function was also discussed and managed carefully. We kept in mind not to invade the hyoid bone laterally so as not to injure the recurrent laryngeal nerve. This procedure is associated with at least 2 disadvantages. Firstly, to our knowledge, no such procedure has been previously reported in the literature; therefore, its clinical response rate and long-term outcome are unknown. Secondly, the procedure is associated with a risk of fistula formation as is common with other surgical procedures performed for lingual thyroid, such as excision via a lateral pharyngotomy technique, because a direct communication is established between the skin of the neck and the pharyngeal cavity [4]. Our patient showed the development of a fistula; however, this resolved within 5 months postoperatively with conservative management.

## Conclusions

Our laryngo fiberscopy-guided suspension procedure is a novel surgical procedure for the treatment of a lingual thyroid. Total preservation of the thyroid tissue, certain resolution of an airway obstruction, and ease and simplicity of the surgical technique are the advantages of this novel procedure.

## Abbreviations

CT: Computed tomography
TSH: Thyroid stimulating hormone
US: Ultrasonography
